# Tamoxifen Reduces Breast Cancer Recurrence in Women with DCIS Who Underwent Mastectomy

**DOI:** 10.3390/curroncol33020089

**Published:** 2026-02-02

**Authors:** Netchanok Sae-sim, Norasate Samarnthai, Warapan Numprasit

**Affiliations:** 1 Division of Head Neck and Breast Surgery, Department of Surgery, Faculty of Medicine Siriraj Hospital, Mahidol University, Bangkok 10700, Thailand; netchanokpum@gmail.com; 2 Department of Pathology, Faculty of Medicine Siriraj Hospital, Mahidol University, Bangkok 10700, Thailand; norasatesmthai@yahoo.com

**Keywords:** DCIS, endocrine treatment, postmastectomy recurrence, contralateral breast recurrence

## Abstract

Background: Adjuvant tamoxifen reduces recurrence in patients with ER-positive DCIS treated with lumpectomy and radiation, but its benefit after mastectomy remains unclear. Methods: We retrospectively analyzed 287 patients who underwent mastectomy for pure DCIS at Siriraj Hospital between 2008 and 2017. Recurrence risk factors were assessed using log-rank test, and survival probabilities were estimated with Kaplan–Meier analysis. Results: Of 180 patients with hormone receptor (HR)-positive pure DCIS treated with mastectomy, 120 (66.7%) received tamoxifen, while the remaining 60 (33.3%) did not. The median follow-up was 8.07 years (0.05–13.8 years). Sixteen (8.9%) recurrences were identified, with 5 in the tamoxifen group and 11 in non-endocrine-therapy (ET) group. The 10-year recurrence-free survival (RFS) was 94.7% in the tamoxifen group compared with 77.9% in the non-ET group. Patients with HR-positive DCIS treated with tamoxifen following mastectomy had significantly less subsequent breast cancer (HR = 0.178; *p* = 0.001). Conclusions: Recurrence of breast cancer after mastectomy for DCIS is rare; however, it carries a high mortality rate for those who relapse. Adjuvant tamoxifen after mastectomy demonstrated a significant reduction in the risk of recurrence in ER-positive DCIS. This study supports the decision to prescribe adjuvant ET in patients with DCIS who underwent mastectomy.

## Simple Summary

This retrospective cohort study evaluated the impact of adjuvant endocrine therapy on recurrence risk in patients with estrogen receptor (ER)-positive ductal carcinoma in situ (DCIS) treated with mastectomy. While tamoxifen is known to reduce subsequent breast cancer in patients with ER-positive DCIS undergoing breast-conserving surgery with radiation, its benefit after mastectomy remains uncertain. Among 180 patients with hormone-receptor-positive DCIS, those who received adjuvant tamoxifen had a significantly higher 10-year recurrence-free survival (RFS) rate than those who did not receive endocrine therapy (94.7% vs. 68.7%). These findings suggest that adjuvant tamoxifen provides a meaningful reduction in recurrence risk and improves long-term outcomes in patients with ER-positive DCIS after mastectomy.

## 1. Introduction

Ductal carcinoma in situ (DCIS) is a noninvasive breast cancer characterized by malignant epithelial cells confined to the ductal system without evidence of stromal invasion [1]. It is most commonly detected by screening mammography and currently accounts for approximately 20–25% of newly diagnosed breast cancer cases [2,3]. Owing to widespread screening, the incidence of DCIS has increased substantially over recent decades [4]. DCIS is generally associated with an excellent prognosis, with reported 20-year breast cancer-specific mortality rates of approximately 3.3% [5]. Nevertheless, a subset of patients remains at risk for local and distant recurrence, including progression to invasive disease. Recent clinical trials have explored active surveillance and omission of surgery for selected low-risk DCIS cases, suggesting that nonoperative management is feasible in carefully selected patients; however, these approaches require longer follow-up before widespread adoption [6]. Despite the relatively favorable outcomes associated with DCIS, the risk of both ipsilateral and contralateral breast cancer events remains clinically relevant and warrants careful long-term management [7,8].

Standard treatment options for DCIS include mastectomy or breast preservation surgery (BCS) with or without radiation therapy (RT) and endocrine treatment, particularly in patients with positive estrogen receptor (ER) [9]. Mastectomy is the treatment of choice in women with multicentric or diffuse DCIS or women who refuse BCS or postoperative RT. The rate of local recurrence (LR) after mastectomy (3%) is lower than that of BCS (13–25%) at 10-year follow-up, with the latter developing invasive in approximately 50% and in situ in 50% [10,11]. Thus, patients with DCIS who are treated with mastectomy and are generally considered cured by surgery and often forgo adjuvant treatments. However, most recurrences after mastectomy are upstage to invasive, which affects the overall prognosis [10,11].

Adjuvant endocrine therapy (ET) is thought to decrease the risk of breast cancer events in the ipsilateral and contralateral breast. In the National Surgical Adjuvant Breast and Bowel Project B-24 (NSABP B-24), a randomized controlled study of tamoxifen following lumpectomy and radiation for DCIS demonstrated that the benefits of tamoxifen were primarily observed in patients with ER/PR-positive DCIS. Over 14.5 years of follow-up, tamoxifen for 5 years significantly reduced any breast cancer events by 42% and any contralateral breast cancer events by 50% [12]. The UK/ANZ DCIS trial found no additional benefit of tamoxifen in patients with DCIS who underwent BCS and RT. However, among patients with DCIS who declined RT, tamoxifen significantly decreased any ipsilateral breast cancer events by 23%, ipsilateral DCIS by 29%, contralateral invasive breast cancer by 71%, and contralateral DCIS by 78% after a median 12.7 years of follow-up [13]. Although most studies supported the role of ET in DCIS, they were limited to BCS and/or RT, not mastectomy.

The present study aimed to determine risk factors for recurrence after mastectomy for pure DCIS and to identify the clinical effectiveness of adjuvant endocrine therapy in these patients. In addition, we attempted to assess patterns of recurrence and characteristics among these patients who develop recurrent disease.

## 2. Materials and Methods

This study was conducted in accordance with the principles of the Institutional Review Board of Siriraj Hospital (approval number 405/2021). Patient consent was exempted due to retrospective research design. The population comprised adult women (age ≥ 18 years) diagnosed with unilateral, pure DCIS who underwent mastectomy at Siriraj Hospital during a ten-year period (January 2008 to December 2017). To maintain the specificity of the cohort, several exclusion criteria were applied: we omitted patients with concurrent invasive breast malignancies, a history of invasive cancer at any site, or contralateral DCIS. Furthermore, cases involving microinvasive DCIS, male patients, and those with distinct histological subtypes, such as lobular carcinoma in situ or Paget’s disease, were excluded. Patients managed with breast-conserving surgery were likewise ineligible for inclusion. For the subset of patients who experienced disease recurrence, comprehensive clinical data were extracted through a systematic review of electronic medical records.

Clinicopathological data were collected. All pathological reports were reviewed to confirm the diagnosis of pure DCIS. The following data were extracted: tumor size, nuclear grade, comedonecrosis, resection margins, ER and PR status, breast reconstruction and adjuvant treatment. Tumor size was not classified due to the lack of an accurate size in pathology reports (32.8%, 59/180). The estrogen receptor (ER) and progesterone receptor (PR) status were defined as positive if ≥1% of tumor cell nuclei were stained or negative if <1%. Recurrence was defined as evidence of disease relapse >6 months after initial treatment of DCIS.

The application of adjuvant ET was at the physicians’ discretion, based on their experience and the patient’s general condition. Additionally, ET was suggested for patients with ER- and/or PR-positive disease at the standard dose of 20 mg orally daily.

Patients who received tamoxifen were scheduled for clinical follow-up every six months for a total duration of five years. In contrast, the group not taking tamoxifen underwent biannual check-ups for two years and then once a year until year five years. Mammographic screening was performed annually, except in cases where prior imaging showed no suspicious findings that warranted closer surveillance.

The primary study objective was the assessment of breast cancer events, defined as biopsy-confirmed DCIS or invasive recurrence localized to the ipsilateral chest wall or contralateral breast. Diseases occurring outside the contralateral breast were categorized as distant recurrences and required verification via clinical examination, radiological imaging, or histological analysis. To evaluate prognostic factors, a comparative analysis was performed between patients experiencing recurrence and those remaining disease-free. Recurrence-free survival (RFS) was calculated as the interval from the date of initial diagnosis to the first documented recurrence.

Data analysis was conducted using SPSS software, version 21.0. Categorical variables were evaluated through the chi-square test or Fisher’s exact test, as appropriate. For continuous data, the Student’s *t*-test was utilized for normally distributed variables, while the Mann–Whitney U test was applied for nonparametric distributions. Survival data, specifically RFS, were calculated using the Kaplan–Meier method and evaluated via the log-rank test. To identify independent predictors of RFS, a multivariate Cox proportional hazards regression model was applied. Statistical significance was defined as a two-tailed *p*-value < 0.05.

## 3. Results

### 3.1. Clinical Characteristics

**Figure 1:**
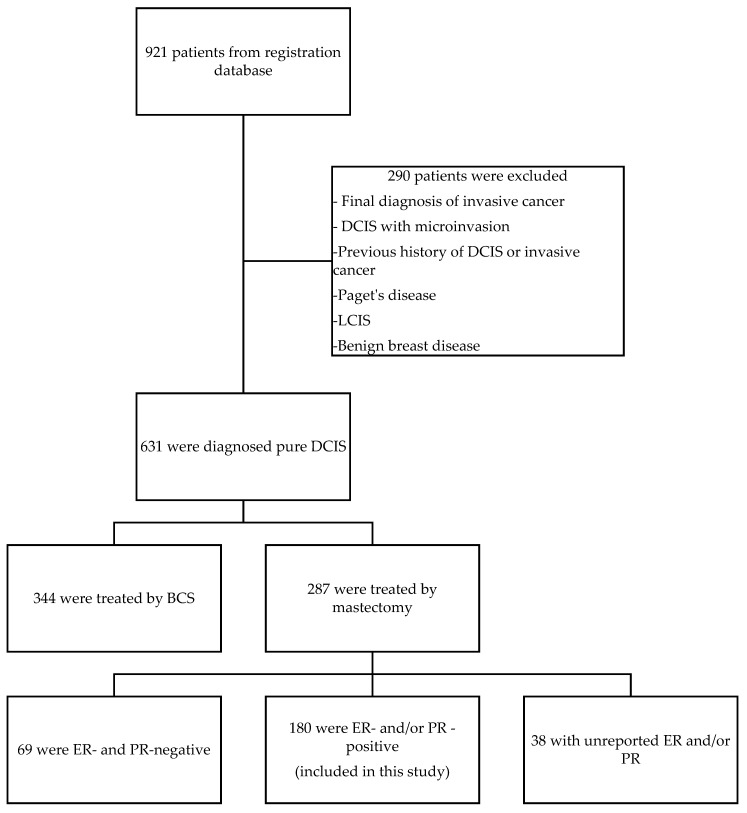
Consort diagram of the study population, showing the starting population, reasons for attrition, and the final groups used for the analysis.

This study enrolled 180 patients with pure DCIS that underwent mastectomy and were ER/PR-positive (Figure 1). Among the 180 patients, 120 patients received adjuvant tamoxifen, while the remaining 60 patients received mastectomy alone. The study population was stratified into two cohorts based on the therapeutic intervention received. A summary of clinicopathologic features and treatment variables is provided in Table 1. Comparative analysis revealed that patients managed with mastectomy alone exhibited no statistically significant difference in tumor grade, surgical margin width, or rate of breast reconstruction when compared to the cohort receiving adjuvant postmastectomy tamoxifen. However, the mastectomy-only group demonstrated a significantly higher prevalence of comedonecrosis compared to the tamoxifen-treated group (*p* < 0.044). The median duration of tamoxifen treatment was 60 months (range, 1 to 111 months); 94 out of 120 patients (78%) received tamoxifen to 5 years.

### 3.2. Comparison of Recurrence-Free Survival

**Figure 2:**
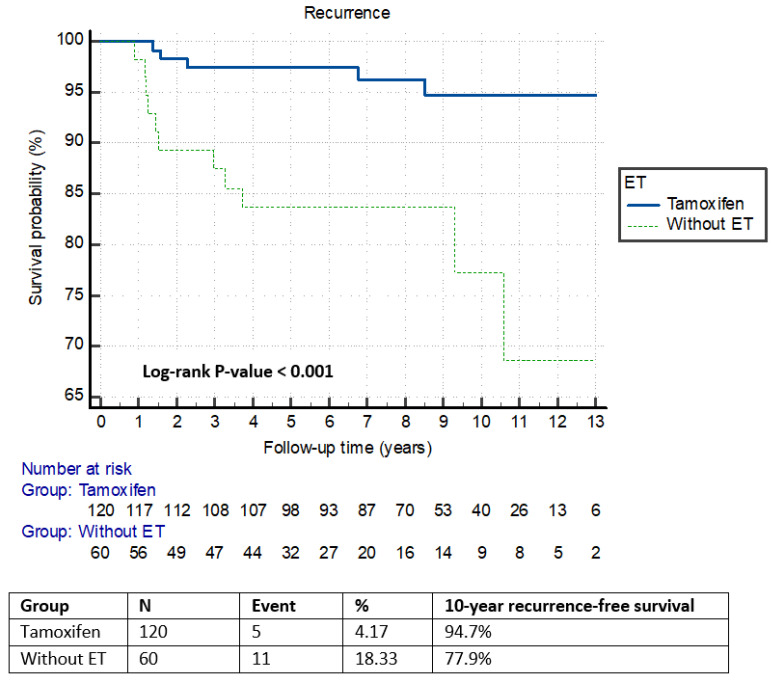
Kaplan–Meier plot of cumulative recurrence-free survival between tamoxifen and without ET in patients with HR-positive DCIS treated with mastectomy.

The mean follow-up time was 8.07 years (range, 0.05 to 13.8 years). Recurrence occurred in 16 patients: 2 patients in the ipsilateral chest wall, 11 patients in the contralateral breast, and 3 patients in distant sites. Fourteen cases had a pathological report of recurrence, of which 9 cases were invasive and 5 cases pure DCIS. Three patients who developed distant metastasis had no evidence of synchronous ipsilateral or contralateral carcinoma, and all died from breast cancer during follow-up. None of the patients had axillary relapse. The median time to recurrence was 1.93 years, and 75% of the patients recured at 6 years. The proportion of patients who developed recurrence was higher in the nonadjuvant ET groups (*n* = 11) and thus had a lower 10-year RFS rate (77.9%; 95% CI, 63.4–92.1) compared to the adjuvant ET group (94.7%; 95% CI, 89.9–99.4) (log-rank *p* < 0.001). Kaplan–Meier curves comparing tamoxifen and non-ET groups demonstrated that the reduction in subsequent breast cancer was restricted to patients with HR-positive DCIS treated with tamoxifen (Figure 2).

### 3.3. Factors Associated with Recurrence-Free Survival

Clinicopathological factors correlated with recurrence were the detection method and the adjuvant ET (Table 2). Univariate analysis showed that only endocrine therapy and detection methods were significant predictors of RFS, while the other factors, including age at diagnosis, ER/PR status, DCIS size, nuclear grade, comedonecrosis, margin status, and immediate breast reconstruction, were not significantly associated with recurrence. Next, a multivariate analysis was performed, including variables with *p* < 0.1 from the univariate analysis. In this model, both the detection methods and adjuvant ET remained independent factors of RFS (Table 3).

### 3.4. Recurrence Patterns and Characteristics

In the mastectomy-alone group, contralateral recurrence was found in eight patients, ipsilateral chest wall recurrence was found in two patients, and distant metastasis was found in one patient. In the adjuvant ET group, five patients had recurrence (three patients had contralateral recurrence, and the other two patients had distant metastasis). The characteristics of 16 patients who developed recurrence are shown in Table 4. The pattern of recurrence did not differ between the DCIS group receiving adjuvant ET and the group without adjuvant ET. The patterns and pathological characteristics of recurrent diseases are summarized in Table 5.

## 4. Discussion

This study focused on patients with hormone-receptor-positive DCIS treated with mastectomy. The results showed that the overall postmastectomy recurrence rate was approximately 8.9%. Additionally, the 10-year recurrence-free survival (RFS) rate was significantly higher in the group receiving adjuvant endocrine therapy (ET) (94.7%) than in the group that did not receive adjuvant ET (68.7%). Multivariate analysis identified adjuvant ET as an independent factor of improved RFS (HR = 0.181, *p* = 0.002), while the method of detection was also significantly associated with RFS (HR = 3.324, *p* = 0.042).

In this study, the recurrence after mastectomy of patients with DCIS was consistent with a previous report, which was around 1–8.1% [14,15]. Although overall recurrence was low in patients with DCIS treated with mastectomy, some studies reported values as high as 16% in patients with high-risk characteristics (margin < 2 mm with high-grade disease and/or comedonecrosis) [16]. In addition, a large population-based study conducted in England found that the incidence of contralateral invasive breast cancer (6.8%) was higher than that of ipsilateral invasive breast cancer (2.8%) [17]. Similarly, this study showed that the highest recurrence occurred contralaterally (11 in 16 patients). The median time to recurrence in this study (1.93 years) was shorter than that reported in most other studies. This pattern may reflect the characteristics of the recurrence group, such as a younger median age (47 years), which, although not statistically significant, is a known risk factor for recurrence in DCIS [18,19]. Additionally, unmeasured factors such as family history or genetic predisposition, as well as possible variability in the quality of breast imaging, may have contributed to earlier recurrences, although these were not assessed in this study.

Adjuvant endocrine treatment was a protective factor for recurrence; on the other hand, symptomatic at presentation increased the risk of recurrence after mastectomy in patients with DCIS. Other risk factors are contradictory in the literature, such as young age, high nuclear grade, close or positive margins, and tumor size, yet none of these were statistically significant in the current study [20,21].

Available data suggest that endocrine therapy administered as adjuvant therapy is beneficial in terms of reducing ipsilateral and contralateral breast events in patients with ER-positive DCIS who underwent BCS [13,22]. However, there are no prospective studies or RCTs that evaluate the benefit, cost-effectiveness, and survival advantage of adjuvant ET in postmastectomy settings; therefore, individual consideration of risks and benefits must be discussed. In our study, those receiving adjuvant tamoxifen had a higher 10-year RFS than patients who did not receive endocrine therapy. Although the median follow-up time was significantly longer in the tamoxifen group (8.52 years vs. 5.94 years, *p* < 0.001), the recurrence rate remained lower, supporting a potential protective effect of tamoxifen, even in patients generally considered at low risk after mastectomy. The effectiveness of tamoxifen is partly influenced by adherence to treatment. A systematic review of early breast cancer demonstrated that non-adherence or non-persistence to adjuvant endocrine therapy negatively impacted event-free survival [23]. Although there are no direct studies on DCIS, our study suggests that the low recurrence rate observed in the tamoxifen group is attributable to high adherence among our patients, with 78% completing the full 5-year course of tamoxifen.

Concerns regarding tamoxifen-related side effects, particularly uterine cancer, thromboembolic events, are discussed in detail with all patients before initiating therapy [24,25]. In the present study, no cases of uterine cancer or thromboembolic complications were reported during the follow-up period. While these risks are well-documented, they remain relatively low, and proper patient selection, counselling, and monitoring can help minimize adverse outcomes. As such, we emphasize the importance of individualized decision making that considers both the benefits in recurrence risk reduction and the potential side effects of tamoxifen.

Following BCS treatment for DCIS, the National Comprehensive Cancer Network (NCCN) guidelines advise scheduling physical examinations every 6 to 12 months for the first five years, followed by annual check-ups [9]. However, specific recommendations for surveillance after mastectomy in this context are not currently offered. In our series, the median crude time from initial treatment to invasive recurrence was approximately 1.93 years; therefore, an annual clinical follow-up physical examination is warranted.

The limitations of our study include its retrospective design and the small number of patients with recurrence. Furthermore, the size and extent of DCIS could not be included in our analysis due to variability in reporting and inconsistencies in quantification parameters, resulting in 32.8% of cases (59 out of 180 patients) lacking this information. Additionally, a relatively short median time to invasive recurrence of 1.93 years raises the possibility that some invasive cancers may have been missed during the initial pathological evaluation. The potential for underdiagnosis is an acknowledged limitation of this study and highlights the challenges in accurately assessing DCIS and its progression. Most patients who developed recurrence had heterogeneously dense breasts, a factor known to reduce the sensitivity of conventional imaging. During the study period, institutional surveillance protocols primarily relied on mammography and ultrasound, with double reading performed selectively in cases with equivocal or questionable imaging findings, while breast MRI was not routinely performed. Consequently, some lesions may have been occult on conventional imaging. Although MRI was not routinely utilized, contralateral breast lesions identified during follow-up had not been evident on prior surveillance mammography or ultrasound. Importantly, most recurrent cases were asymptomatic and detected through imaging during routine surveillance, with the exception of patients who subsequently developed distant metastasis.

Our study highlights that adjuvant endocrine treatment can be beneficial, even in low-risk breast cancer cases such as patients with DCIS postmastectomy. However, it is particularly valuable in selected cases with a high risk of relapse. Factors that may contribute to underestimating the risk of recurrence, even after mastectomy—such as close tumor margins or large DCIS lesions that might obscure small invasive foci—should be carefully considered when determining the need for additional adjuvant treatment.

## 5. Conclusions

Retrospective analyses showed a significant benefit in RFS of adjuvant tamoxifen in patients with HR-positive DCIS after mastectomy. These data should be considered when counseling a patient after mastectomy for DCIS. The use of adjuvant ET can be considered for selected patients; the undesirable adverse effects of ET and the patient’s quality of life must be considered during individual decision making. The present study provides information to offer an additional therapeutic option that physicians and patients should consider in patients with DCIS treated with mastectomy.

## Figures and Tables

**Table 1:** Clinicopathologic and treatment characteristics (total number of patients = 180).

Variables	Overall *n* = 180 (%)	Tamoxifen *n* = 120 (%)	No Adjuvant ET *n* = 60 (%)	*p* Value *
Age at initial surgery, years				
Median (range)	52 (24–79)	52 (27–79)	51 (24–79)	0.798
Method of detection				
Screening detected	93 (51.7)	63 (52.5)	30 (50)	0.752
Clinical examination	87 (48.3)	57 (47.5)	30 (50)	
ER				
Negative	10 (5.6)	4 (3.3)	6 (10)	0.086
Positive	170 (94.4)	116 (96.7)	54 (90)	
PR				
Negative	16 (8.9)	9 (7.5)	7 (11.7)	0.354
Positive	164 (91.1)	111 (92.5)	53 (88.3)	
Nuclear grade of DCIS				
Low	17 (9.5)	11 (9.3)	6 (10)	0.687
Intermediate	76 (42.2)	53 (44.2)	23 (38.3)	
High	87 (48.3)	56 (46.7)	31 (51.7)	
Comedonecrosis				
Present	49 (27.2)	27 (22.5)	22 (36.7)	0.044
Absent	36 (20)	22 (18.3)	14 (23.3)	
Unreported	95 (52.8)	71 (59.2)	24 (40.0)	
Resection margin width				
≤2 mm	23 (12.8)	13 (10.8)	10 (16.7)	0.269
>2 mm	157 (87.2)	107 (89.2)	50 (83.3)	
Immediate breast reconstruction				
Yes	42 (23.3)	29 (24.2)	13 (21.7)	0.709
No	138 (76.7)	91 (75.8)	47 (78.3)	
Recurrence				
Yes	16 (8.9)	5 (4.2)	11 (18.3)	0.002
No	164 (91.1)	115 (95.8)	49 (81.7)	

* Pearson chi-square, exact sig

**Table 2:** Clinical characteristics between recurrence and non-recurrence groups.

Variables	Recurrence		*p* Value
	Yes (*n* = 16)	No (*n* = 164)	
Age at initial surgery, years			
Median (range)	48 (24–79)	52 (27–79)	0.179
Method of detection			
Screening detected	4 (25.0%)	89 (54.3%)	0.025
Clinical	12 (75.0%)	75 (45.7%)	
ER			
Negative	0	10 (6.1%)	0.603
Positive	16 (100%)	154 (93.9%)	
PR			
Negative	1 (6.3%)	15 (9.1%)	1.0
Positive	15 (93.8%)	149 (60.9%)	
Size (mm) ^a^	22.0 (6.8, 47.5)	20.0 (12.0, 31.0)	0.942
Nuclear grade of DCIS			
Low	2 (12.5%)	15 (9.1%)	0.374
Intermediate	8 (50.0%)	68 (41.5%)	
High	6 (37.5%)	81 (49.4%)	
Comedonecrosis			
Present	5 (31.3%)	44 (26.8%)	0.743
Absent	4 (25.0%)	32 (19.5%)	
Unreported	7 (43.8%	88 (53.7%)	
Resection margin width			
≤2 mm	3 (18.8%)	20 (12.2%)	0.436
>2 mm	13 (81.3%)	144 (87.8%)	
Immediate breast reconstruction			
Yes	5 (31.3%)	37 (22.6%)	0.535
No	11 (68.8%)	127 (77.4%)	
Endocrine therapy			
Tamoxifen	5 (31.3%)	115 (70.1%)	0.002
No	11 (68.8%)	49 (29.9%)	

^a^, expressed as median (P25, P75), using Mann–Whitney U test. Tumor size data were available for 14 of 16 recurrence and 107 of 164 non-recurrence cases.

**Table 3:** Univariate and multivariate analysis of RFS for the whole cohort.

Variable	Univariate Analysis	Multivariate Analysis	*p* Value
	HR (95% CI)	HR (95% CI)	
Tamoxifen	0.178 (0.061–0.516)	0.181 (0.063–0.527)	0.002
Detection methods during clinical examination	3.338 (1.076–10.351)	3.324 (1.042–10.037)	0.042

Abbreviations: RFS = recurrence-free survival; HR = hazard ratio; CI = confidence interval (based on ***p***-value in the univariate analysis).

**Table 4:** Clinicopathological characteristics of 16 patients who developed recurrence.

Patient	Age at Diagnosis	Method of Detection DCIS	Grade	Comedonecrosis	Margin	Size (mm)	Immediate Reconstruction	Received Tamoxifen	Duration of ET	Time to Relapse (Years)	Method of Recurrence Detection	Site of Relapse	Breast Density	Relapse Histology	Status at the Last Follow-Up	Interval from Relapse to Last Follow-Up (Years)
1	52	Mass	2	Absent	Negative	15	No	Yes	18.4	1.4	Screening	Contralateral	Heterogeneous	DCIS	Alive NED	4.67
2	33	Mass	2	Present	Close anterior	63	Autologous	Yes	18.4	1.6	Symptom	Liver	Heterogeneous	Invasive	Death	0.63
3	79	Mass	2	Absent	Negative	20	No	Yes	4.1	2.3	Symptom	Bone	Almost entirely fat	Unknown	Death	2.6
4	63	Discharge	2	Unknown	Negative	4	No	Yes	34.8	8.5	Screening	Contralateral	Heterogeneous	Invasive	Alive NED	3.36
5	57	Screening	3	Unknown	Negative	11	No	Yes	60.9	6.8	Screening	Contralateral	Heterogeneous	Invasive	Alive NED	5.24
6	69	Mass	2	Unknown	Negative	Unknown	No	No	0	1.5	Screening	Contralateral	Almost entirely fat	Invasive	Alive NED	6.28
7	44	Screening	1	Unknown	Negative	6	Autologous	No	0	1.2	Screening	Contralateral	Heterogeneous	DCIS	Alive NED	9.25
8	44	Screening	3	Present	Negative	7	No	No	0	1.2	Screening	Contralateral	Extreme	Invasive	Alive NED	4.35
9	42	Screening	3	Present	Close anterior	45	Combine	No	0	0.9	Screening	Contralateral	Heterogeneous	DCIS	Alive NED	3.11
10	46	Discharge	2	Absent	Negative	55	Autologous	No	0	3.3	Screening	Ipsilateral	Heterogeneous	Invasive	Alive NED	2.22
11	63	Mass	2	Present	Negative	29	No	No	0	3.7	Screening	Contralateral	Heterogeneous	Invasive	Alive NED	0.94
12	24	Discharge	2	Absent	Negative	5	No	No	0	2.9	Screening	Contralateral	Heterogeneous	DCIS	Alive NED	1.66
13	49	Mass	1	Unknown	Negative	24	No	No	0	9.3	Screening	Contralateral	Heterogeneous	Invasive	Alive NED	3.51
14	41	Mass	3	Unknown	Negative	30	No	No	0	1.5	Symptom	Liver + Bone	Heterogeneous	Unknown	Death	0.75
15	52	Mass	3	Unknown	Negative	Unknown	No	No	0	10.6	Screening	Contralateral	Heterogeneous	Invasive	Alive NED	1.65
16	28	Mass	3	Present	Close anterior	60	Implant	No	0	1.2	Screening	Ipsilateral	Extreme	DCIS	Alive NED	1.89

**Table 5:** Summarized patterns and histology of recurrence (*n* = 16).

Variables	Overall *n* = 16	Tamoxifen *n* = 5 (%)	No Adjuvant ET *n* = 11 (%)	*p* Value *
Median follow-up time (years)	7.96	8.52	5.94	<0.001
Recurrence patterns				
Ipsilateral	2	0	2	0.396 *
Contralateral	11	3	8	
Distant metastasis	3	2	1	
Histology of recurrence				
DCIS	5	1	4	1.000
Invasive ductal carcinoma	9	3	6	
Unknown	2	1	1	

* Pearson chi-square, exact sig.

## Data Availability

The datasets used and/or analyzed during the current study are available from the corresponding author on reasonable request.
